# Supplementary material for: Content-rich biological network constructed by mining PubMed abstracts
Source: BMC Bioinformatics. 2004 Oct 8;5:147. doi: 10.1186/1471-2105-5-147 (PMC528731; doi:10.1186/1471-2105-5-147)
Supplement: Additional File 2 — The original results of the above study (non-essential files are deleted to keep the file size under the limit set by BMC bioinformatics). [file 1471-2105-5-147-S2.bz2 › chilibotAdditionalFile2/dip05/257ID8242751E1020/html/CCND1_CDKN1A.html]

 


 **CCND1** and **CDKN1A** 
  
Found 633 abstracts in PubMed, retrieved 05.  
 

 What does Google say? 
 PDF only 
| .edu only 

---

**Interactive relationship** (e.g. stimulation, inhibition, etc)

**Non-interactive relationship** (e.g. studied together, co-existance, homology, etc.)

- In addition, the latter induces changes in gene expression by up regulating p53, Waf 1  [ **CDKN1A** ] , Cas, Dap kinase and caspases and 14 and down regulating Bcl  [ **CCND1** ]  2.  Ref: 12887065 Biol Chem, 2003
- Changes in the expression of caspase 3, bax, bcl  [ **CCND1** ]  2 and p21cip waf  [ **CDKN1A** ]  were assessed by quantitative Western blotting.  Ref: 12888913 Int J Oncol, 2003
- The combination effectively up regulated caspase 3 and bax and down regulated bcl  [ **CCND1** ]  2 and p21cip waf  [ **CDKN1A** ] .  Ref: 12888913 Int J Oncol, 2003
- These events were accompanied by down regulation of the Raf 1 MEK ERK pathway as well as diminished expression of Bcr Abl and cyclin D1  [ **CCND1** ] , cleavage of p21 CIP1  [ **CDKN1A** ]  and pRb, and induction of the stress related kinases JNK and p38 MAPK.  Ref: 12893773 BloodBlood, 2003
